# Favorable outcomes for patients with refractory systemic lupus erythematosus treated with rituximab as evidenced with a follow-up of ≥ 10 years: a real-world evidence study

**DOI:** 10.1007/s00296-025-05879-3

**Published:** 2025-04-28

**Authors:** Chrysanthi Staveri, Chrysa Lykoura, Konstantinos Melissaropoulos, Stamatis-Nick C. Liossis

**Affiliations:** 1 Division of Rheumatology, 1st Floor, Patras University Hospital, 26504 Rion, Patras, Greece; 2 Division of Rheumatology, “Agios Andreas” General Hospital, Patras, Greece; 3 Division of Rheumatology, Department of Internal Medicine, University of Patras School of Medicine, Patras, Greece

**Keywords:** Systemic lupus erythematosus, Lupus nephritis, B cell, Rituximab, Refractory systemic lupus erythematosus, Proteinuria

## Abstract

Treatment of Systemic Lupus Erythematosus (SLE) remains challenging. The aim of this real-world evidence study of patients with refractory SLE treated with rituximab (RTX) was to explore for any potential long-term effect(s) of this particular B cell depletion approach. This study included patients with SLE who had i) received at least 1 cycle of RTX and had ii) an at least 10 yr of follow-up after their first RTX infusion. Outcomes were assessed at 1 year and at their latest evaluation that was ≥ 10 yr after RTX treatment initiation. In cases where the SLE Disease Activity Index 2000 (cSLEDAI-2 k) was employed, a response was defined as a cSLEDAI-2 k of less than 4 in cases where the cSLEDAI-2 k was ≥ 4. In cases where the cSLEDAI-2 k was 2–4 at baseline, a response was defined as a cSLEDAI-2 k of 0. RTX was administered in 62 patients with SLE. For this real-world evidence study 23 patients (25 cases) with SLE (all Caucasian female, age range: 14–72 yr, mean: 31 yr) with active or relapsing disease, fulfilling inclusion criteria were enrolled. RTX treatment was associated with a response rate of 68.75% after 1 yr and 75% after ≥ 10 yr. The median cSLEDAI-2 K score decreased from 5.83 ± 3.70 at baseline to 1.95 ± 2.40 (*p* < 0.001) at 1 yr and to 2.37 ± 3.00 (*p* < 0.001) at the ≥ 10 yr time-point of follow-up. Our data suggest that RTX may indeed represent an alternative therapeutic option in patients with SLE refractory to standard treatment with an acceptable safety profile.

## Introduction

Systemic lupus erythematosus (SLE) is a highly heterogenous systemic autoimmune disease with an unclear etiopathogenesis. B cells are thought to play a central role in the cascade of immune-mediated events characterizing SLE [1–4]. B cells are persistently hyperactive, produce autoantibodies (autoAbs) and they also act as antigen presenting cells to T cells [5, 6]. B cells from patients with SLE also secrete cytokines and express or overexpress toll-like receptors implicated in B cell activation [7, 8]. Inadequate therapeutic management of active SLE is not uncommon; however, this is associated with an increased risk of organ damage and morbidity or even mortality. Targeting B cells in SLE seems plausible, firstly due to the numerous SLE B cell abnormalities described and secondly, due to the efficacy of B cell-targeting treatments. The rationale of B cell targeting in SLE has been previously extensively reviewed [9–14].

Rituximab (RTX) is an anti-CD20 chimeric monoclonal antibody (mAb). CD20 is expressed on the surface membrane of most B cells except pro-B cells and plasma cells. RTX causes a transient depletion of mature B cells from the stage of pre-B cells to the stage of memory B cells. A couple of double-blind, placebo-controlled trials of RTX in patients with SLE and in patients with lupus nephritis (LN) failed to show superiority over placebo [15, 16]. Despite that, both the American College of Rheumatology (ACR) and the European Alliance of Associations for Rheumatology (EULAR) guidelines recommend that RTX can be introduced in patients with both resistant LN and refractory SLE [17, 18]. According to Kidney Disease: Improving Global Outcomes (KDIGO) guidelines RTX treatment may be considered in patients with Class III and Class IV LN showing persistent disease activity or inadequate response to initial standard-of-care therapy and also as an immunosuppressive treatment in patients with Class V LN [19]. The estimated off-label employment of RTX treatment in Europe ranges from 0.5% to 1.5% of patients with SLE, possibly reflecting limitations set by local authorities [20].

The aim of this real-world evidence study was to address the question if RTX treatment could result in long-term favorable effect(s) in patients with refractory SLE, suggesting that mAb-mediated B cell depletion at some point could potentially alter the natural course of the disease in the long run, in patients with SLE.

## Methods

### Study design

This is a real-world evidence study of patients with SLE treated in 2 tertiary rheumatology centers of Southwestern Greece. For our study, inclusion criteria were: (a) patients had to fulfill SLE classification criteria [21], (b) patients had to be treated with administration of at least one cycle of RTX, (c) patients had completed an at least 10-year follow up evaluation period following their initial RTX infusion.

### Outcome measures

Response to RTX treatment was assessed at: (i) 1 yr, and (ii) at the latest clinical evaluation that was ≥ 10 years following initial RTX infusion. Failure or lack of clinical response was defined as no improvement of clinical manifestations or laboratory parameters. In cases where the SLE Disease Activity Index 2000 (cSLEDAI-2 k) was employed, a response was defined as a cSLEDAI-2 k < 4 post-treatment when the cSLEDAI-2 k was > 4 at baseline. In cases where the cSLEDAI-2 k was 2–4 at baseline, a response was defined as achieving a cSLEDAI-2 k of 0.

For patients with LN a complete response was defined as a proteinuria of < 500 mg/24 h and an estimated glomerular filtration rate (eGFR) of $\ge$ 60 ml/min. A partial response was defined as a reduction of proteinuria of > 50% compared to baseline values and an eGFR of $\ge$ 60 ml/min. Regarding lung involvement a response was defined as an FVC decline of ≤ 5% predicted values. Any improvement(s) of the imaging findings on CT scans of the chest was also considered as a response.

### Statistical analysis

Statistical analysis was performed using the paired Student’s *t*-test. *P*-values < 0.05 were considered as statistically significant.

## Results

Our first patient with LN was treated with RTX in 2002, and subsequently a total of 62 patients with SLE have been treated with RTX at some time point during the course of their disease. For this cross-sectional study 23 female patients (25 cases) with SLE (age range: 14 – 72 yr, mean: 31 yr) with active or relapsing disease, fulfilling inclusion criteria were enrolled. The median disease duration was 6 yr (range: 2 mo-27 yr) at the time of the first RTX treatment infusion. Our patient population demographics and clinical features are presented in Table 1.

**Table 1:** Demographic and clinical features of patients at the time of RTX administration

Pt no	Age (years)	Sex	Disease duration (years)	Previous treatments	Disease manifestations
1	34	F	4	CS, HCQ, MMF, CYC	Nephritis, rash
2	23	F	2	CS, HCQ	Neutropenia
3	50	F	20	CS, IVIG, AZA, CYC,	Bowel vasculitis
4	16	F	5	CS, CYC	Shrinking lung syndrome
5	21	F	5	CS, AZA, CYC, IVIG, HCQ	Lupoid hepatitis
6	44	F	6	CS, CYC	Neuromyelitis optica
7*	14	F	2 months	CS, HCQ	Benign intracranial hypertension
			7	CS, HCQ, MMF, plasmapheresis	Thrombotic thrombopenic purpura
			7	CS, HCQ, CYC, MMF	Nephritis
8	32	F	27	CS, HCQ,	rash, interstitial pneumonitis
9	31	F	3	CS, AZA, MMF, CYC	Nephritis
10	32	F	11	CS, CYC, IVIG	Arthritis, shrinking lung syndrome
11	14	F	2	CS, HCQ	Nephritis
12	22	F	6	CS, MTX	Stomatitis
13	23	F	3	CS, HCQ	Nephritis, rash
14	25	F	1	CS, MMF	Diplopia
15	23	F	8	CS, HCQ, MTX	Arthritis Autoimmune hemolytic anemia
16	30	F	3	CS, HCQ	Arthritis
17	31	F	3	CS, MTX, AZA, CYC	Arthritis, camptocormia
18	24	F	2	CS, CYC, IVIG, MMF	Nephritis
19	25	F	6 months	CS, HCQ	Arthritis
20	37	F	3	CS, HCQ, MTX, AZA	Arthritis, livedo reticularis
21	72	F	1	HCQ	Autoimmune hemolytic anemia
22	37	F	5	CS, MMF, CSA	Nephritis
23	52	F	5	HCQ, AZA, MMF, CYC, CS	Nephritis

*CS* corticosteroids, *HCQ* hydroxychloroquine, *MTX* methotrexate, *AZA*: azathioprine, *IVIG*: intravenous immunoglobulin, *MMF* mycophenolate mofetil, *CYC* cyclophosphamide, *CSA* cyclosporine A. * Patient 7 was treated with RTX for 3 different disease manifestations at 3 different time points (included as 3 cases).

The serological profile of the patients enrolled is presented in detail in Table 2. All patients tested positive for ANA. Anti-dsDNA auto Ab were positive in 7/23 patients, anti-Ro were positive in 10/23 patients, anti-RNP in 9/23 patients, and anti-Sm in 6/23 patients. Depressed circulating concentrations of C3 and/or C4 were found in 14/23 patients at the time of RTX treatment initiation.

**Table 2:** Serological profile of SLE patients

Pt no	ANA	Anti-dsDNA	Anti-Sm	Anti-Ro	Anti-La	Anti-RNP	Anti-Histones	↓ C3	↓ C4
1	1:1280	+	+	–	–	+	–	+	+
2	1:320	–	–	–	–	+	–	+	+
3	1:160	–	–	+	–	–	–	–	+
4	1:1280	–	–	+	+	+	–	+	+
5	1:1280	+	+	+	–	–	+	+	+
6	1:640	–	–	–	–	–	–	–	–
7	1:1280	+	–	–	–	–	–	+	+
8	1:160	–	+	+	–	–	–	–	–
9	1:640	–	–	+	+	–	–	–	–
10	1:1280	+	+	–	–	+	–	+	+
11	1:2560	+	–	–	–	+	–	+	+
12	1:160	–	–	+	–	–	–	–	+
13	1:160	–	–	+	–	–	–	+	–
14	1:1280	+	–	–	–	+	–	+	+
15	1:640	–	+	–	–	+	–	+	+
16	1:1280	–	–	+	–	+	–	–	–
17	1:320	–	–	+	–	–	–	+	+
18	1:160	+	–	–	–	–	–	+	+
19	1:1280	–	–	+	–	–	–	+	+
20	1:320	–	–	–	–	–	–	+	–
21	1:160	–	+	–	–	–	–	–	–
22	1:160	–	–	–	–	–	–	–	–
23	1:2560	–	–	–	–	+	–	–	–

Clinical manifestations at the time of RTX introduction included LN in 8 patients, arthritis in 6, neuropsychiatric involvement in 4, vasculitis in 2, lung involvement in 3 and hematological abnormalities in 3. RTX was also administered in one corticosteroid-plus-azathioprine (AZA) resistant case of lupus hepatitis and in a plasmapheresis-plus-steroid resistant case of thrombotic thrombopenic purpura. Some patients either simultaneously belonged into more than one of the above clinical manifestations groups or they had different clinical features at different time points during the course of their disease.

Previous treatments included corticosteroids in all patients, hydroxychloroquine (HCQ), mycophenolate mofetil (MMF), AZA, methotrexate (MTX), cyclophosphamide (CYC), intravenous immunoglobulin (IVIG) and cyclosporine A (CSA). The commonly used RTX regimens: “the RA protocol”, which was administered at a dose of 1000 mg repeated after 2 weeks or “the lymphoma protocol” (4-weekly infusions of 375 mg/m^2^) were randomly employed.

Response was assessed 1 yr after the first cycle of RTX treatment and during the latest visit (≥ 10 yr following first cycle of RTX). The response rate was 68.75% at 1 yr. The response rates at the ≥ 10 yr timepoint was 75%. Subsequently it was asked the question if certain SLE manifestations were more or less prone to respond to RTX treatment. The responses seen at 1 yr and at ≥ 10 yr after RTX treatment initiation according to isolated SLE manifestations is analyzed below.

### Lupus nephritis

This study aimed to identify the effect(s) of RTX treatment in patients with LN after 1 yr and after ≥ 10 yr enrollment. The laboratory parameters of the patients with LN are presented in Table 3. Among patients with LN (8 cases), proteinuria ranged from 1,062 mg/24 h to 11,500 mg/24 h. An active urinary sediment was evident in 3 out of 8 patients before RTX treatment introduction.

**Table 3:** Laboratory parameters of the patients with lupus nephritis at baseline and 1-yr outcomes

Pt no	Creatinine (mg/dL)	eGFR (ml/min)	Urine protein (mg/24 h)	Active urine sediment	Response in 1 year
1	0.5	126	2,744	No	Complete
13	0.7	105	1,200	No	Complete
11	0.9	94	2,200	Yes	Complete
18	0.8	105	11,500	Yes	Partial
9	2.7	18	1,210	No	No, dialysis
7	0.7	130	5,280	No	Partial
22	1.4	50	4,250	No	No, dialysis
23	0.5	113	1,062	Yes	Complete

A renal biopsy was not performed (although recommended) in 5 patients for other-than-medical reasons. Renal biopsy was compatible with Class III LN in 1 patient, Class V in a second patient, and Class IV & Class V in a third. The latter patient achieved a partial response after RTX introduction and underwent a second kidney biopsy. Histology of her second biopsy was compatible with predominately Class V LN with the appearance of sclerotic lesions. Among patients with LN, evidence of interstitial nephritis was present in all 3 biopsies. Finally, one patient developed end-stage renal disease, another one developed chronic kidney disease while the third patient responded clinically to RTX treatment.

**Fig. 1:**
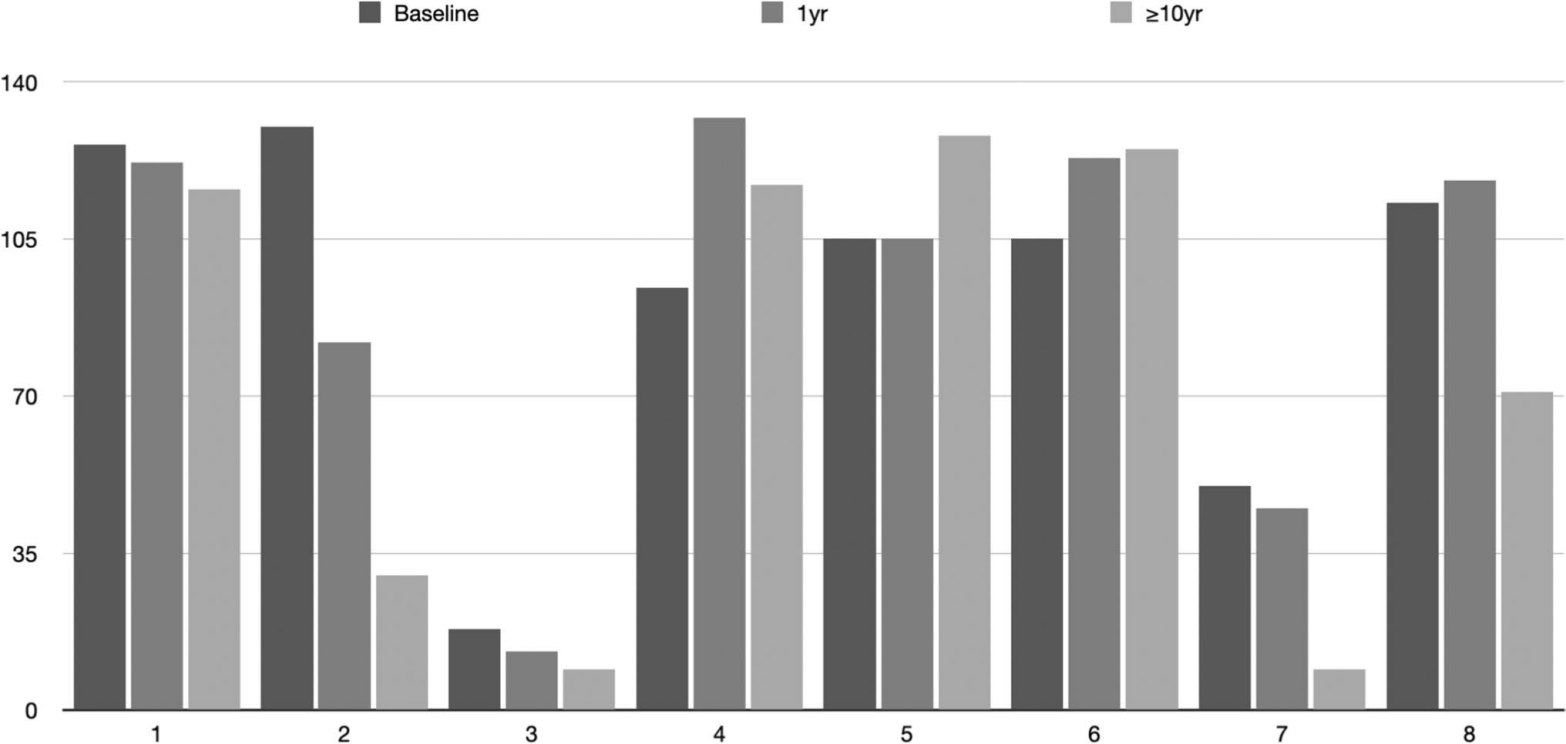
Renal function (eGFR) of patients with lupus nephritis at baseline, after 1 year and after ≥ 10 years following RTX treatment introduction compared to baseline values [X axis: patients’ numbers, Y axis: eGFR (ml/min)]

The effects of RTX treatment on renal function were examined by evaluating the eGFR before RTX introduction (Mean ± SD: 92.62 ± 38.95), 1 yr after RTX treatment initiation (Mean $\pm$ S.D.: 92.50 $\pm$ 42.85, *p* = 0.99 vs. baseline) and finally after ≥ 10 yr (Mean $\pm$ S.D.: 75.62 $\pm$ 52.78, *p* = 0.47 vs. baseline) (Fig. 1). Therefore, eGFR did not decline significantly after 1 or after more than 10 years of follow-up. It is of note that non-responders (2/8) that developed end-stage renal disease (ESRD) had an already seriously limited kidney function at baseline (eGFR: 18 ml/min and 50 ml/min, respectively).

After 1 yr, an overall renal response was observed in 75% (6 of 8); a complete response in 50% (4 of 8) and a partial response in 25% (2 of 8). This beneficial renal outcome dropped to 62.5% during the ≥ 10 yr visit. More specifically, 1/2 patients achieving an initial (1 yr) partial response was finally classified as having a complete response at the ≥ 10 yr visit. Of note, 2 out of the 3 patients who did not eventually benefit from RTX treatment developed ESRD necessitating hemodialysis; these were the 2/8 patients who did not respond at the earlier (1 yr) timepoint. Those 2 patients along with a third patient who finally developed chronic kidney disease were defined as non-responders. Thus, patients with LN of this study did benefit from RTX treatment because their eGFR apparently stabilizes early after RTX initiation and this effect lasts long. It should be noted herein that the long-term analysis of kidney function of patients with LN treated with the standard-of-care has been associated with less favorable long-term results. In contrast, RTX did not prevent the development of ESRD in those patients having an eGFR < 60 ml/min at the time of RTX treatment introduction.

### Non-renal SLE

To evaluate the potential effect(s) of RTX treatment in refractory non-renal SLE manifestations the short and long-term efficacy of B cell depletion in 17 additional cases were analyzed.

#### Neuropsychiatric manifestations

This study included 4 patients with neuropsychiatric involvement to estimate the benefit(s) of RTX treatment in SLE patients with neuropsychiatric SLE (NPSLE). Their clinical manifestations included: benign intracranial hypertension, diplopia, camptocormia due to diffuse dystonia and transverse myelitis. The overall clinical response was 100% after 1 yr of RTX treatment introduction. The one patient with transverse myelitis relapsed at a later-than-1 yr timepoint. Therefore, the overall clinical response was 75% at ≥ 10 yr. Even though the number of patients is small, our long-term observations suggest that RTX may be of benefit in patients with NPSLE, a manifestation of SLE with obscure pathogenesis and incompletely defined treatment approach.

#### Hematological manifestations

The efficacy of RTX treatment in SLE patients with hematological abnormalities was also examined. Cytopenias were recorded in 3 patients (neutropenia in 1 and autoimmune hemolytic anemia in 2). An increased neutrophil count from 663/mm3 to 1,188/mm3 at 1 yr, and to 1,581/mm3 after ≥ 10 yr was recorded in the patient with neutropenia. The first patient with autoimmune hemolytic anemia (Ht: 30%, Hb: 9.4 g/dL) improved at 1 yr (Ht: 37.8%, Hb: 12.8 g/dL), but relapsed afterwards (Ht: 27%, Hb: 8.4 g/dL). The second patient with autoimmune hemolytic anemia, that was accompanied by arthritis, (Ht: 22.0%, Hb: 7.2 g/dL) also improved after RTX treatment introduction (Ht: 32.3%, Hb: 9.5 mg/dL and Ht: 32.6%, Hb: 10.2 mg/dL at 1 yr and ≥ 10 yr, respectively). Therefore, the hematological response were 100% at 1 yr and 66.66% after ≥ 10 yr, which could be considered as encouraging, despite the small number of patients enrolled in the study.

#### Vasculitic manifestations

To address the efficacy of RTX treatment in other severe non-renal manifestations of SLE 2 patients with vasculitis were included. There was 1 patient with intestinal vasculitis presenting with abdominal pain and vomiting. Contrast enhanced CT-scans of the abdomen demonstrated bowel wall thickening and abnormal wall enhancement (“double-halo” or “target” sign). A relapse occurred at 1 yr after RTX treatment initiation as well as at a later time point before 10 yr. Both relapses were successfully managed with IV pulsed corticosteroids. Another patient presenting with livedo reticularis resistant to corticosteroid treatment displayed complete resolution not only after 1 yr but also after ≥ 10 yr. Overall, the clinical response was 50% at 1 yr and 50% at the ≥ 10 yr timepoint.

#### Pulmonary manifestations

Aiming to examine the efficacy of RTX treatment in SLE pulmonary disease, 3 patients with lung involvement were enrolled. Among those patients 1 out of 3 had chronic lupus pneumonitis manifesting as interstitial lung disease. High-resolution computed tomography (HRCT) of the chest showed that a previously mild and restricted interstitial fibrosis changed to / evolved into organizing pneumonia at 1 yr after RTX treatment introduction; however, after ≥ 10 yr all CT scan findings had cleared. RTX was also administered in 2 patients with “shrinking lung” syndrome. The first patient did not benefit from RTX treatment at any timepoint (FVC: 67.2% predicted at baseline, 57.4% predicted at 1 yr and 58% predicted at ≥ 10 yr), but the second patient improved not only at 1 yr but also at the ≥ 10 yr visit (FVC: 50% predicted at baseline, 62% at 1 yr and 60% at ≥ 10 yr). The overall clinical response was 33.33% at 1 yr and 66.67% at the ≥ 10 yr timepoint. Thus, RTX treatment might represent an alternative therapeutic approach in patients with SLE and lung involvement, although the number of patients enrolled in the study is limited.

#### Mucocutaneous manifestations

This study evaluated 4 patients with mucocutaneous involvement, including erythematosus lesions in 3/4 and oral ulcers in 1/4 in order to determine the short- and long-term results of RTX treatment on skin and mucosal involvement. In all cases cutaneous disease was persistent and refractory to corticosteroids, topical therapies and antimalarials. A relapse occurred at 1 yr after RTX treatment in 1 patient despite an initial impressive improvement. However, this same patient had no signs of active skin disease at the ≥ 10 yr timepoint. There was no benefit from RTX treatment in one patient at all. Thus, the clinical response rates were 50% and 75% at 1 yr and at the ≥ 10 yr timepoint, respectively, rendering RTX treatment another potentially useful therapeutic option in the management of refractory mucocutaneous disease in patients with SLE, although the number of patients included in the study is small.

#### Articular manifestations

In order to evaluate the impact of RTX treatment in SLE patients with refractory articular involvement 6 patients with treatment-resistant arthritis were enrolled. All of them had persistent polyarthritis principally affecting the wrists, metacarpophalangeal and proximal interphalangeal joints, causing significant functional impairment. A combination of previously administered DMARDs such as HCQ and MTX were of no benefit. An initial improvement was recorded in all 6 patients; however 2/6 patients relapsed at 1 yr after RTX administration; both patients improved following a short-term corticosteroid course. Therefore, the clinical response was 66.67% at 1 yr after RTX treatment initiation. However, none of our 6 patients had active arthritis at the ≥ 10 yr timepoint. Thus, the long-term clinical response for SLE arthritis was 100%. In conclusion, RTX treatment may represent a reliable long-term treatment solution for patients with SLE and refractory arthritis, despite the limited number of patients enrolled in the study.

#### Miscellaneous manifestations

There were 2 patients with distinct manifestations that could not be classified into previous groups. A patient with lupus hepatitis [aspartate aminotransferase (AST): 308, alanine aminotransferase (ALT): 187, gamma-glutamyl transferase (GGT): 101, alkaline phosphatase (ALP): 221 at baseline] improved after a single course of RTX treatment showing complete normalization of serum values of the liver enzymes (AST: 33, ALT: 41, GGT: 45, ALP: 65 at 1 yr and AST: 18, ALT: 10, GGT: 13, ALP: 76 after ≥ 10 yr).

A patient with thrombotic thrombopenic purpura [hematocrit (Ht): 8.8%, hemoglobin (Hb): 3.9 g/dL, presence of schistocytes in the peripheral blood, platelets (PLT): 46,000/mm^3^ at baseline) responded favorably to RTX treatment (Ht: 35.8%, Hb: 11.8 g/dL, PLT: 252,000/mm3 at 1 yr, and Ht: 30.2%, Hb: 10.2 g/dL, PLT: 296,000/mm3 at the ≥ 10 yr timepoint). The overall short- and long-term therapeutic response for those isolated miscellaneous manifestations was 100%. 

### The cSLEDAI-2 k index response

**Fig. 2:**
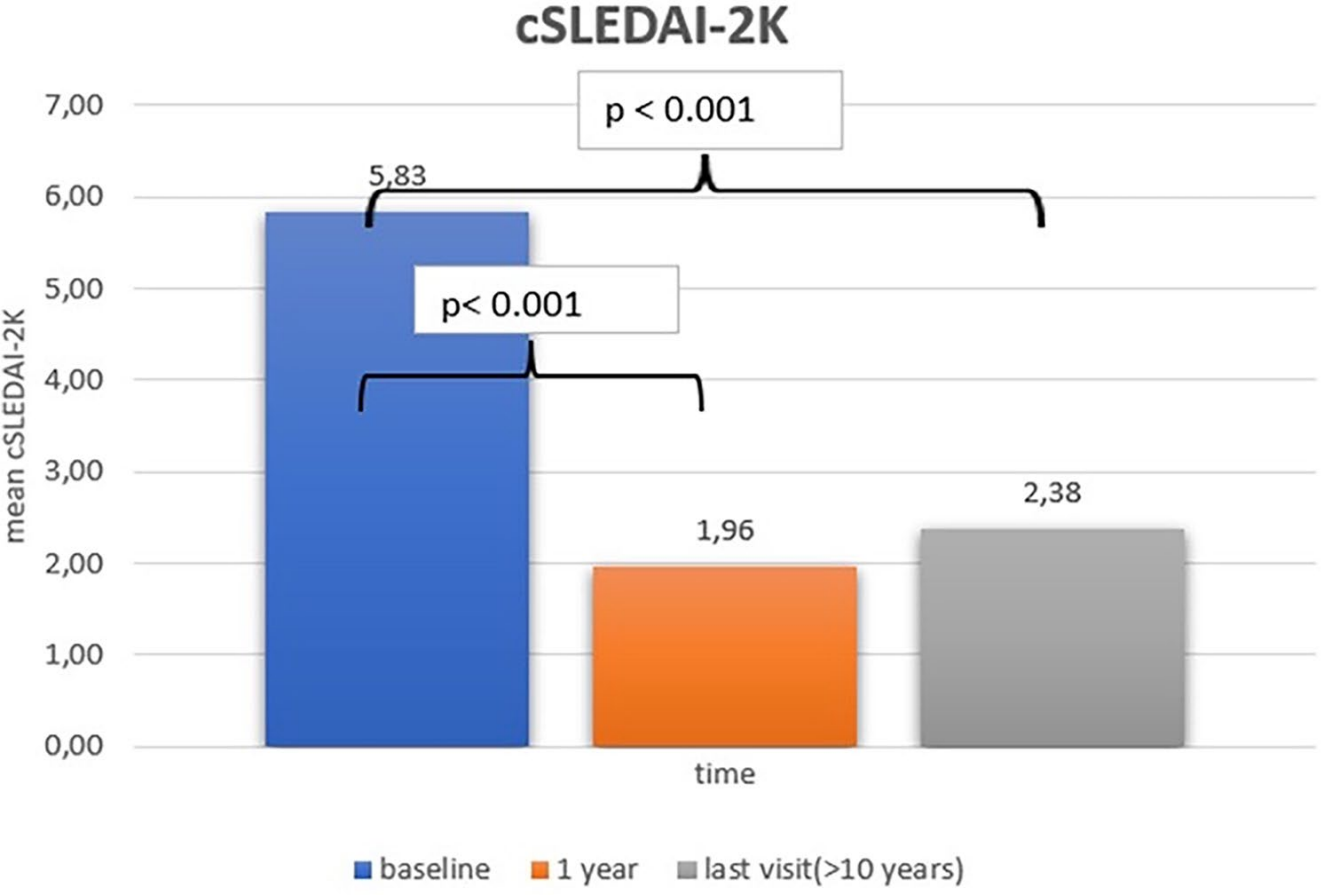
Reduction of cSLEDAI-2 k at 12 months after RTX treatment initiation and at last visit compared to baseline (*p* < 0.05)

It was decided that 16 cases were suitable for cSLEDAI-2 k calculation. According to the results the median cSLEDAI-2 K score decreased from 5.83 ± 3.70 at baseline to 1.95 ± 2.40 (*p* < 0.001) at 1 yr after RTX treatment initiation and to 2.37 ± 3.00 (*p* < 0.001) at the ≥ 10 yr time point of follow-up (Fig. 2).

### Follow-up

Over the course of our at least 10 yr of follow-up, 10 out of 23 of our patients relapsed (43.48%) (Table 4). The earliest relapse was observed at 6 months and the latest one at 13 years after initial RTX treatment introduction. Among patients that relapsed 8 were retreated with RTX and 3/8 (37.50%) re-responded. Notably, 1 patient developed diffuse cervical lymphadenopathy after 1 month following the first RTX infusion. Lymph node biopsy excluded malignancy and DNA analysis disclosed clonal TCR-rearrangements but not malignancy; B cells were practically absent from the lymph node, a finding attributed to the preceding treatment.

**Table 4:** Follow-up

Pt no	Follow up (years)	RTX treatment repetition	Reason for RTX treatment repetition	Time to first relapse
1	10	No		
2	16	2	Relapse	6 months
3	10	2	Relapse	2 years
4	13	1	Remission maintenance	
5	13	5	Relapse	1 year
6	13	7	Remission maintenance	
7	14	7*	Improvement maintenance for the first 4 years	4 years
8	10 (death)	No (worsening of the rash)		
9	10	1	Relapse	6 months
10	13	8	Relapse	2 years
11	14	2	Relapse	5 years
12	13	No		
13	16	No		
14	10	No		
15	15	5**	Relapse	1 year
16	12	No		
17	14	1	Remission maintenance	
18	14	3	Remission maintenance	
19	12	No		
20	10	2	Relapse	6 months
21	10	multiple	Relapse	4 years
22	12	multiple	Relapse	6 months
23	14	multiple	Remission maintenance	

*This patient retreated with RTX for remission maintenance of lupus nephritis and not for benign intracranial hypertension or TTP, **This patient retreated with RTX due to arthritis and not due to autoimmune hemolytic anemia

Multiple cycles of RTX treatment were employed successfully for remission maintenance in 6 out of 23 patients. However, 3/23 (13%) patients achieved a sustained remission after the administration of one single course of RTX treatment.

Causes for RTX treatment discontinuation were: lack of efficacy (9/23, 39.13%) and adverse events (4/23, 17.39%). Three patients were lost to follow-up after completing a 10 yr follow-up. However, it should be noted that RTX treatment was discontinued in 6/23 (23.08%) because of long-term SLE remission, based on clinical judgement.

### Steroid sparing effect

**Fig. 3:**
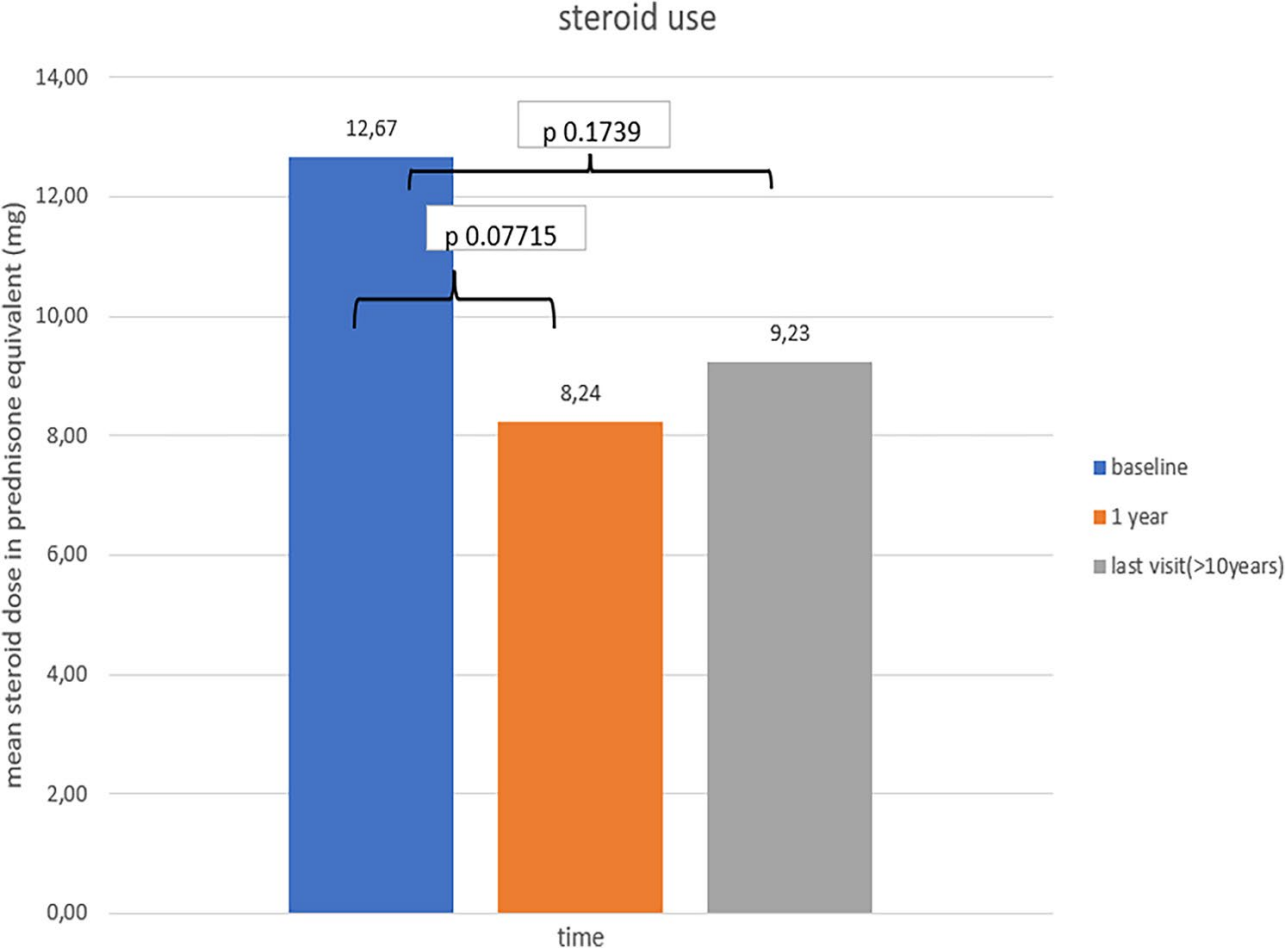
Median corticosteroid dose at 12 months after RTX treatment initiation and at last visit compared to baseline

The potential effects of RTX treatment on the reduction of the mean daily dose of corticosteroids were analyzed in 22 out of 25 cases where data were available. The mean daily dose of corticosteroids was reduced both at the1yr and at the ≥ 10 yr time points; however, such decreases were not but statistically significant (*p* = 0.07 and *p* = 0.17, respectively) (Fig. 3).

In conclusion, RTX treatment in our cohort was not associated with a significant steroid sparing effect. The importance of long-term low doses of steroids and the contribution of other-than-steroids agents to achieve this has been recently recognized in all therapeutic trials in SLE.

### Safety

No cases of mycobacterial or systemic fungal infections occurred during the ≥ 10 yr follow-up. Uncomplicated herpes zoster infection developed in 2 patients. A patient developed CMV-related retinitis resulting in single-eye blindness 3 yr following the single RTX infusion cycle she received. This same patient developed ecthyma gangrenosum due to Pseudomonas aeruginosa and Fusobacterium necrophorum co-infection 4 yr after RTX treatment, leading to hospitalization and a prompt response after prolonged IV antibiotic treatment. Due to the occurrence of unusual and severe infections despite a long time-interval from RTX treatment in this patient, laboratory tests were performed that revealed diffuse hypogammaglobulinemia. Detailed evaluation of previously unavailable to us medical records found that she had long-established diffuse hypogammaglobulinemia and a flow-cytometrically documented circulating B cell deficiency. Severe infections were not unknown to her previously, although she was never offered IVIG treatment. Despite treating her with regular IVIG substitution infusions for years afterwards in our center, the patient finally succumbed due to SARS-CoV-2 infection 10 yr after her one and only RTX infusion cycle.

Mild infusion reactions were recorded in 4 patients. A single case of late-onset neutropenia was identified in our cohort, with the absolute neutrophil count dropping to < 500/mm^3^ 2 months after the second course of RTX treatment. This patient presented with low-grade fever without any other signs of infection and was not hospitalized; she was successfully treated with a short course of granulocyte-colony stimulating factor.

## Discussion

This real-world evidence study analyzed the efficacy and safety of RTX treatment over a period of at least 10 years in 23 patients (25 cases) with SLE refractory to standard treatment. Overall, the addition of RTX in the treatment regimen was associated with a favorable outcome in 68.75% after 1 yr and 75% after ≥ 10 yr. Based on the results of the long-term follow-up of our cohort, RTX could be considered as an alternative therapeutic approach for patients with SLE refractory to standard-of-care treatment such as corticosteroids and immunosuppressive drugs, including CYC and MMF.

Several studies have underscored the efficacy of RTX treatment in patients with SLE resistant to standard of care treatment [22–34]. Published studies are very heterogeneous regarding the numbers of patients enrolled (ranging from 261 to 18), the SLE manifestations treated (ranging from LN only to overall treatment-resistant manifestations), the treatment cycles (ranging from 1 RTX cycle to multiple), the follow-up duration (ranging from 6 to 77mo), the evaluation tools employed to determine treatment responses [such as SLEDAI, British Isles Lupus Assessment Group (BILAG), renal responses only and “overall clinical judgment”] and even the RTX-treatment scheme (in most it was RTX only but in one was with CYC co-administration). Reported results cannot be directly compared to our data, mostly because the evaluation time-points are remarkably different (6–77 mo) from the ones employed in our report (1 yr and ≥ 10 yr). However, a favorable outcome that is quite similar to our data has been reported in most of previously reported studies. A potential exception may be the low percentage of responses published from the German registry of the Autoimmune Diseases that report a “complete response” in 46.8% and a “partial response” in 34.2% of patients at a mean follow-up of almost 10 mo. However, such response categorization was based on physician clinical judgment only. Although the authors also report a significant decline of a baseline SLEDAI of 12.3 to 3.3, percentages of patients achieving such a response are lacking. Of note also is the study of LN-only patients treated in the RITUXILUP trial (a steroid-sparing regimen consisting of 2 doses methylprednisolone 500 mg and RTX 1 g, 2 weeks apart followed by MMF maintenance treatment alone) [32]. Results on RTX effectiveness in this trial are the highest of all reported above (90% by 37 weeks and 85% by 52 weeks). Moreover, when the follow-up was extended to 77mo remission maintenance was preserved along with a preservation of renal function [34]. This study differs from ours not only because it included only patients with LN, but, perhaps more importantly, because RTX was administered early as a first treatment approach and not later, following the unsatisfactory results of previous immunosuppression as the LN patients we included. Moreover, such results may imply that RTX treatment might result in better renal outcomes when administered earlier but not later in the disease course.

According to the results of a Bayesian meta-analysis, risks of ESRD in patients with LN decreased between the 1970s and the mid-1990s and then remained stable, but ESRD risk increased in the late 2000s [35]. Despite extensive use of immunosuppressive treatments through the 2000s, a reduction in ESRD risks was not confirmed.

The beneficial effect of RTX treatment in our study was not limited to patients with LN only. Patients with articular involvement, hematological abnormalities and neuropsychiatric SLE also improved after RTX treatment administration (66.67%, 100%, 100% at 1 yr and 100%, 66.67%, 75% at ≥ 10 yr, respectively) (Table 5).

**Table 5:** RTX-induced clinical response at 12 months and at last visit, based on SLE manifestations

Organ involvement	% of response (12 months)	% of response (last visit)
Neuropsychiatric	100%	75%
Hematological	100%	66.67%
Vasculitis	50%	50%
Articular	66.67%	100%
Mucocutaneous	50%	75%
Pulmonary	33.33	66.67%
Miscellaneous	100%	100%
Nephritis	75%	62.5%

The number of patients/cases enrolled in our report is limited. However, to our opinion one strength of our study is that our cohort represents exclusively resistant-to-treatment patients. Perhaps more importantly though, our follow-up period is long; per inclusion criteria, patient’s follow-up not only equals but almost always exceeded 10 yr and it extended to up to 16 yr. To our knowledge, this is the first report of such a long-term (at least 10 yr) follow-up of patients with SLE treated with a B cell depleting agent. Importantly also, RTX was administered in various clinical manifestations of SLE that were difficult to manage and were reluctant to treatment; moreover, in some patients such manifestations were not only severe but even life-threatening**.** Of note, 13% of our patients with resistant SLE who received only a single course of RTX went into full remission; the SLE manifestation treated with RTX never relapsed during the long-term follow-up (≥ 10 yr). In fact, because of that, RTX treatment has been discontinued in these particular patients.

Limitations / disadvantages of our study are: the fact that this is a real-world evidence evaluation of patients evaluated in 2 tertiary care centers of our region; our results do not have the value of double-blind, placebo-controlled trials. The clinical benefits seen in our study cannot be exclusively attributed to RTX because our patients received additional treatments during the long-term follow-up; this limitation of our study is shared with all other previously reported studies since all are based on daily clinical practice and not protocols. In addition, not all patients clinically diagnosed with LN underwent a kidney biopsy; other-than-medical reasons precluded the performance of a kidney biopsy in some of our LN patients.

Avoiding the adverse events of long-term high doses of corticosteroids was one of the reasons for the employment of therapeutic protocols without oral steroids in cases of LN [32]. In our study the achievement of a therapeutic response resulted in an absolute but not statistically significant decrease of the mean daily dose of prednisone at both time points of our evaluation.

Based on the vast amount of experience and published knowledge, although RTX therapy is considered generally safe infections are always a potential matter of concern [36]. It is currently understood that decreased re-population of antibody-forming cells may predispose to hypogammaglobulinemia and subsequent infections. Among our patients only one developed severe infections and eventually died; we cannot ascribe such adverse effects to RTX treatment since both diffuse hypogammaglobulinemia as well as B cell deficiency preexisted.

Some of our patients relapsed at sometime during their follow-up despite an initial improvement. In cases of subsequent disease flare and/or relapse, one might consider a new course of RTX treatment. To this point, our data could be considered as encouraging. However, further particularly randomized controlled clinical trials are necessary to determine the efficacy and safety of RTX treatment in patients with SLE. Studies might also identify which SLE patients subgroups are more suitable to RTX treatment and which subsets might be considered as RTX resistant. Furthermore, our study cannot determine the appropriate dosage to be employed, the optimal duration of therapy, the potential need for re-treatment as well as the appropriate administration of concomitant medications.

Depleting B cells in SLE has eventually emerged as a rather rewarding therapeutic strategy. This is clearly outlined in a recent case series of 11 patients with SLE, where B cell depletion mediated by a single infusion of anti-CD19 chimeric antigen receptor (CAR) T cells, following preconditioning with fludarabine and CYC, was highly effective with an acceptable safety profile for up to 2 yr [37]. In such patients peripheral B cell reconstitution was evident after almost 110 days post-treatment. However, positive therapeutic effects persisted for much longer, suggesting that B cell depletion had a long-lasting-therapeutic effect that was not dependent on the mere presence or absence of B cell per se.

In addition, obinutuzumab, a humanized type II anti-CD20 mAb results in a potent B cell elimination. A recently published study evidenced the significant benefit of obinutuzumab treatment in patients with LN [38]. A post-hoc analysis also suggested that obinutuzumab treatment was associated with a significantly lower risk of renal-related events, eGFR drops and time-to-LN flares [39]. A recent study also suggests that obinutuzumab may also be effectively introduced in SLE patients with both renal and non-renal disease developing secondary unresponsiveness to RTX treatment [40].

RTX causes peripheral B cell depletion and despite its efficacy, it remains B cell non-specific, since it does not specifically target autoreactive B cells only. Even though previous efforts to target autoreactive B cells only were not rewarding, we suggest that future better tailored attempts towards this direction are clearly needed.

## Data Availability

Open data to share.
